# Identification of Programmed Cell Death-related Biomarkers for the Potential Diagnosis and Treatment of Osteoporosis

**DOI:** 10.2174/0118715303326112241021061805

**Published:** 2025-01-08

**Authors:** Yancheng Huo, Meng Guo, Yihan Li, Xingchen Yao, Qingxian Tian, Tie Liu

**Affiliations:** 1 Department of Orthopaedic Surgery, Beijing Chaoyang Hospital, Capital Medical University, Beijing 100020, China

**Keywords:** Osteoporosis (OP), programmed cell death (PCD), bone homeostasis, osteoblast, osteoclast, machine learning, diagnosis, nomogram

## Abstract

**Background:**

Osteoporosis (OP) is a skeletal condition characterized by increased susceptibility to fractures. Programmed cell death (PCD) is the orderly process of cells ending their own life that has not been thoroughly explored in relation to OP.

**Objective:**

This study is to investigate PCD-related genes in OP, shedding light on potential mechanisms underlying the disease.

**Methods:**

Public datasets (GSE56814 and GSE56815) were analyzed to identify differentially expressed genes (DEGs). We employed the least absolute shrinkage and selection operator (LASSO), Boruta, and random forest (RF) algorithms to pinpoint hub PCD-related genes in OP and construct a predictive nomogram model. The performance of the model was validated through ROC curve analysis, calibration curves, and decision curve analysis. Additionally, transcription factor (TF) interaction analysis and functional enrichment analysis were conducted to explore the regulatory networks and biological pathways involved.

**Results:**

We identified 161 DEGs, with 30 prominently associated with PCD. Five hub genes, PDPK1, MAP1LC3B, ZFP36, DRAM1, and MPO, were highlighted as particularly significant. A predictive nomogram integrating these genes demonstrated high accuracy (AUC) in forecasting OP risk, with an AUC of 0.911 in the GSE56815 dataset. The validation confirmed the gene model efficacy in differentiating OP risk and clinical applicability. The subsequent TF-gene interaction analyses revealed that these hub genes are regulated by multiple TFs, indicating their central role in OP pathology. Functional enrichment analysis of the hub genes indicated significant involvement in apoptosis, autophagy, and immune response pathways.

**Conclusion:**

This study identified PDPK1, MAP1LC3B, ZFP36, DRAM1, and MPO as potential biomarkers and proposes a nomogram based on hub genes for predicting osteoporosis risk.

## 1 INTRODUCTION

Osteoporosis (OP) is a prevalent systemic metabolic bone disorder marked by reduced bone mineral density (BMD), deteriorating bone tissue microstructure, and increased bone brittleness, resulting in a higher risk of fracture [1-3]. The gold standard for assessing OP is dual-energy x-ray absorptiometry (DXA), whereby a diagnosis is established for individuals exhibiting BMD levels more than 2.5 standard deviations (SDs) below those of young, healthy adults (T-score ≤ -2.5) [4, 5]. However, referred to as a “silent disease,” OP frequently lacks specific symptoms until a bone fracture occurs [6]. DXA is not prevalent before the occurrence of brittle fractures [7]. Additionally, osteoporotic fractures pose a significant threat to people's health, especially the elderly, and result in substantial social and economic burdens [8]. Unfortunately, early diagnostic and therapeutic biomarkers for OP have not yet been discovered, highlighting the urgent need to identify new biomarkers for the early detection and intervention of OP. Cell death, a fundamental process in biology, is broadly categorized into two main types: accidental cell death (ACD) and programmed cell death (PCD), involving the intricate mechanisms governing cellular fate and homeostasis. PCD encompasses diverse processes such as apoptosis, pyroptosis, necroptosis, cuproptosis, ferroptosis, autophagy-dependent cell death, entotic cell death, parthanatos, netotic cell death, lysosome-dependent cell death, oxeiptosis, and alkaliptosis [9, 10].

Apoptosis is a controlled cell death process that maintains internal environment homeostasis without eliciting inflammation [11]. Pyroptosis involves cell swelling, lysis, and proinflammatory cytokine release *via* inflammasome activation [12]. Necroptosis, regulated by various signaling pathways and dependent on RIP1 kinase activity, exhibits necrotic morphology [13]. Cuproptosis results from copper accumulation, leading to accumulation of proteins and depletion of iron-sulfur cluster proteins [14]. Ferroptosis is characterized by iron-dependent lipid hydroperoxide accumulation [15]. Autophagy-dependent cell death facilitates cellular component renewal [16]. Entotic cell death occurs when one cell engulfs and kills another [17]. Parthanatos is triggered by excessive PARP1 nuclease activity due to oxidative stress-induced DNA damage [18]. Netotic cell death involves neutrophil extracellular trap (NET) release [19]. Lysosome-dependent cell death involves lysosomal membrane permeabilization and cathepsin release [20]. Oxeiptosis, sensitive to reactive oxygen species, mitigates ROS-related inflammation independently of caspase activation [21]. Alkaliptosis is pH-dependent cell death driven by intracellular alkalinization [22].

Bone homeostasis depends on the coordinated activity of osteoblasts derived from bone mesenchymal stem cells (BMSCs), and the osteoclasts generated by the fusion of mononuclear macrophages, and any imbalance in these processes may lead to heightened bone breakdown and decreased bone growth, resulting in various bone diseases, including OP [23]. Moreover, PCD regulates bone metabolism by affecting osteocyte activity, suggesting that targeting PCD regulatory molecules could effectively manage OP. However, the precise processes and signaling routes of PCD in OP still remain mysterious, warranting further research to elucidate the functions of genes and pathways related to PCD. Gene microarray technology and bioinformatics tools offer opportunities to explore PCD-related gene characteristics in OP, facilitating the identification of dependable markers and molecular groups for improved early diagnosis and intervention of diseases.

## 2 MATERIALS AND METHODS

### 2.1 Data Source and Processing

The datasets GSE56814 and GSE56815 [24] were sourced from the Gene Expression Omnibus (GEO) database (http://www.ncbi.nlm.nih.gov/geo/) *via* the GEOquery R package [25]. The GSE56814 dataset contains 42 control and 31 OP samples, while the GSE56815 dataset comprises 40 control and 40 OP samples. Sequencing for GSE56814 utilized the GPL5175 platform and GPL570 for GSE56815, both targeting human peripheral blood samples. After data preparation, the aforementioned datasets were normalized using the SVA package [26] to obtain the resulting dataset, which included 82 control samples and 71 OP samples. Subsequently, the Limma package [27] was utilized to identify the differentially expressed genes (DEGs) by comparing control samples with those from OP samples. The screening thresholds for identifying DEGs were *P*-Value <0.05 and |log2 fold change (FC)| >0.2. The visualization of the results was performed using the ggplot2 package, which generated heat maps and a volcano plot.

### 2.2 Identification of PCD-Related Hub Genes for OP

A total of 1382 genes associated with PCD were collected from a previous study [9]. Furthermore, to identify PCD-related candidate genes for OP, the overlapping genes among the OP DEGs and the PCD-related genes were first identified. Machine learning algorithms, including the least absolute shrinkage and selection operator (LASSO) method with the glmnet package [28], the Boruta method with the Boruta package [29], and the random forest (RF) method with the randomForest package [30], were then utilized to screen for PCD-related hub genes for OP. PCD-related hub genes for OP were chosen based on the shared genes identified by these three methods. Additionally, the expression levels of these genes were assessed using violin plots, and their chromosomal positions were depicted by the RCircos package [31].

### 2.3 Constructing Regulatory Networks of Transcription Factors of the Hub Genes

Transcription factors (TF) play an essential role in gene expression regulation [32]. In our research, we imported the PCD-related hub genes for OP into the online tool NetworkAnalyst (https://www.networkanalyst.ca/, version 3.0) [33] accessed on 19 September 2023 to construct a TF-gene regulatory network. Subsequently, the network was displayed using Cytoscape software (v3.9.2) [34].

### 2.4 Constructing and Validating the Nomogram

The rms R package was utilized to construct a nomogram model for diagnosing OP incidence. The “Total Points” in the model represent the scores assigned to corresponding factors. Receiver operating characteristics (ROC) curve analysis, conducted with the pROC package [35], evaluated the diagnostic capabilities of the hub genes and the nomogram model for OP by calculating the area under the curve (AUC). Subsequently, the predictive ability of the nomogram model was evaluated through a calibration curve. Finally, decision curve analysis (DCA) was applied to assess the practical utility of the model [36].

### 2.5 Consensus Cluster Analysis

Unsupervised hierarchical clustering was applied to the 71 OP samples from the integrated dataset, based on the differences in the expression of the hub genes, using the ConsensusClusterPlus R package [37]. The clustering settings included maxK = 6, clusterAlg = km, and distance = Euclidean.

### 2.6 Evaluating Immune Infiltration

The single sample gene set enrichment analysis (ssGSEA) was performed to measure the relative abundance of 17 immune-related pathways within the OP samples. The enrichment scores were then compared across the sub-clusters, employing the GSVA [38] and GSEAbase [39] R packages.

### 2.7 Enrichment Analysis of PCD-Related Candidate Genes for OP

In order to explore the biological significance of PCD-related candidate genes associated with OP, the clusterProfiler package was employed to conduct Gene Ontology (GO) [40] and Kyoto Encyclopedia of Genes and Genomes (KEGG) analysis [41], where a *P*-value less than 0.05 was regarded as statistically significant. Additionally, to evaluate whether a predefined set of genes demonstrates significant agreement between two biological states, Gene Set Enrichment Analysis (GSEA), a computational approach, was conducted to assess the agreement between a predefined set of genes and two biological states [42]. For this analysis, the reference gene set ‘c5.bp.v7.0.entrez.gmt’ was obtained from the Molecular Signature Database (MSigDB) [43]. A *P*-value below 0.05 was regarded as statistically significant.

### 2.8 Statistical Analysis

All data were examined and visualized using R software (version 4.3.1). Figure panels were assembled using Adobe Illustrator (CC 2020).

## 3 RESULTS

### 3.1 Identification of DEGs

**Fig. (2):**
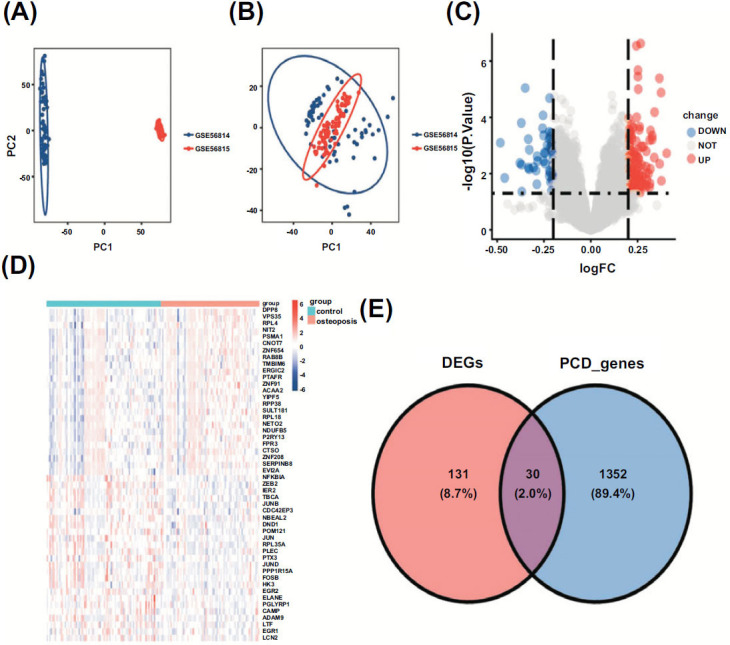
Identification of DEGs. (**A**, **B**) Results from PCA dimensionality reduction before and after data set processing. (**C**) Volcano plot showing all DEGs of OP. (**D**) Heatmap showing the top 50 DEGs. (**E**) The venn diagram showing the overlap of genes between DEGs and PCD.

Fig. (**1**) displays the study's flow diagram. The batch effects were eliminated from the GEO dataset to form the integrated dataset (Figs. **2A** and **B**), comprising 82 control samples and 71 OP samples. A total of 161 DEGs associated with OP were identified, with 114 upregulated and 47 downregulated, based on significance criteria. Figs. (**2C** and **2D**), respectively display the volcano plot and heatmap illustrating DEGs. Fig. (**2E**) shows the Venn diagram of 30 PCD-related candidate genes of OP found in the intersection of DEGs on PCD-related genes.

### 3.2 Identification of PCD-Related Hub Genes in OP

**Fig. (3):**
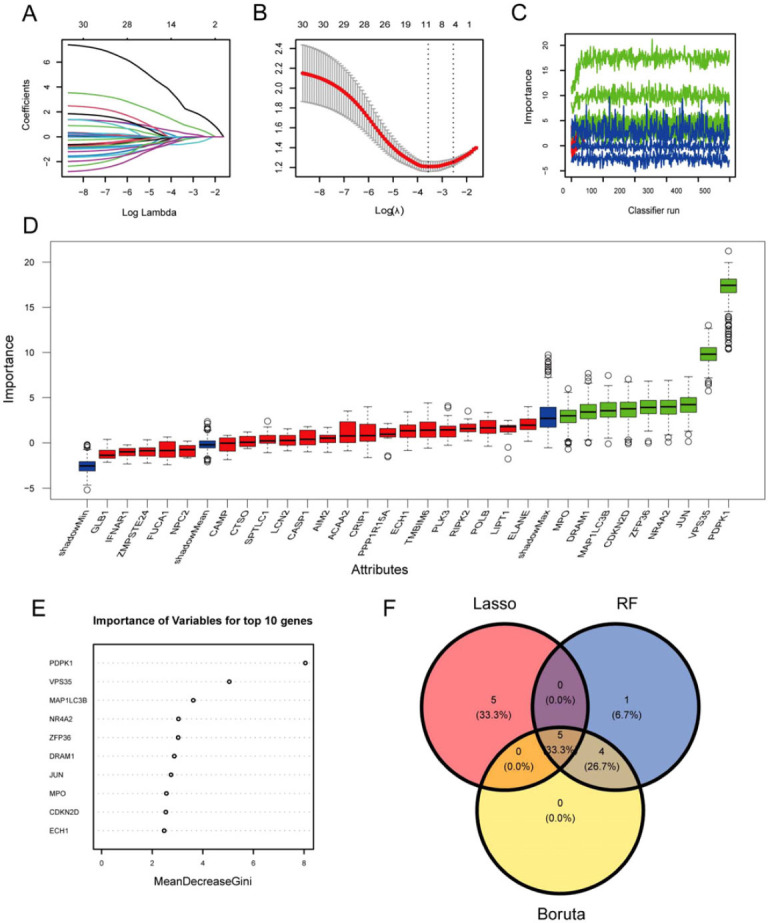
Identification of PCD-related hub genes for OP. (**A**, **B**) Lasso regression analysis results. (**C**, **D**) Feature selection *via* Boruta algorithm. (**E**) Gene importance scores of RF model. (**F**) The venn diagram shows the overlap of candidate genes among the above three algorithms.

The LASSO, Boruta, and RF algorithms were employed to identify characteristic genes with a great impact on OP from the 30 PCD-related candidate genes. The LASSO algorithm identified 10 characteristic genes (Figs. **3A** and **B**), while the Boruta algorithm identified 9 characteristic genes (Figs. **3C** and **D**). Additionally, the RF algorithm identified 10 characteristic genes (Fig. **3E**). Among the overlapping genes selected by the three algorithms, namely PDPK1, MAP1LC3B, ZFP36, DRAM1, and MPO, these were designated as the hub genes (Fig. **3F**).

**Fig. (4):**
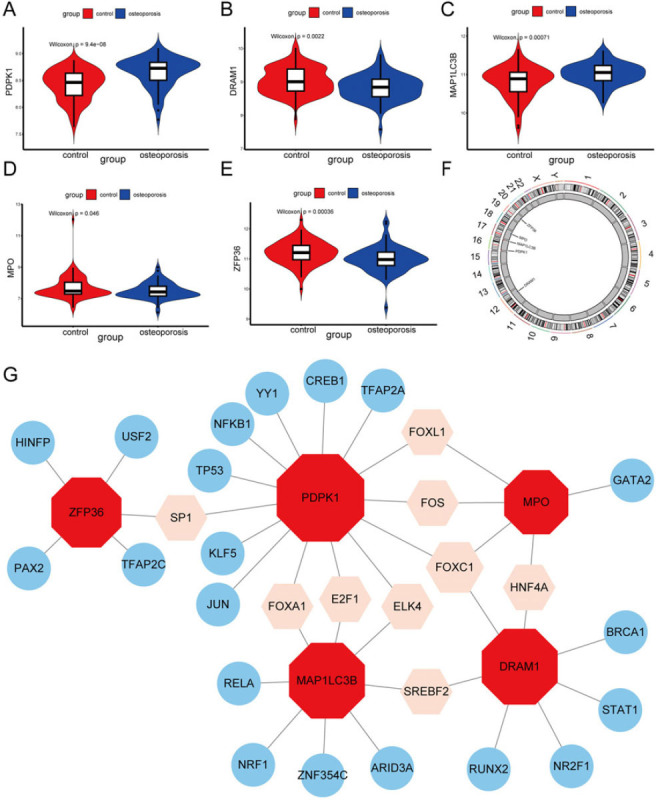
Expression and Regulatory Analysis of PCD-related hub genes for OP. (**A**-**E**) Violin plots showing the expression levels of PCD-related hub genes for OP. (**F**) The location of PCD-related hub genes for OP on chromosomes. (**G**) TF-gene regulatory network of PCD-related hub genes for OP. Red nodes represent hub genes, and nodes of other colors represent TFs.

Violin plots of the expression levels of PCD-related hub genes revealed that PDPK1 and MAP1LC3B were significantly higher in OP samples compared to controls, while DRAM1, MPO, and ZFP36 showed significantly lower expression levels in OP samples (Figs. **4A**-**E**). The positions of PCD-related hub DEGs were annotated on human chromosomes (Fig. **4F**).

### 3.3 TF-Gene Interaction Network of PCD-Related Hub Genes

Furthermore, to reveal the regulatory mechanisms governing hub genes, we employed network analyst to predict the TFs that interact with the five hub genes. Subsequently, we employed cytoscape to visualize the TF-gene regulatory network, which encompassed 34 nodes and 35 edges, with each hub gene being subject to regulation by multiple TFs. Notably, PDPK1 exhibited regulation *via* 14 TFs, MAP1LC3B was regulated by eight TFs, and DRAM1 was regulated by seven TFs. Furthermore, we identified nine TFs capable of regulating more than one hub gene, with FOXC1 capable of regulating three genes simultaneously (Fig. **4G**).

### 3.4 Performance of PCD-Related Hub Genes

**Fig. (5):**
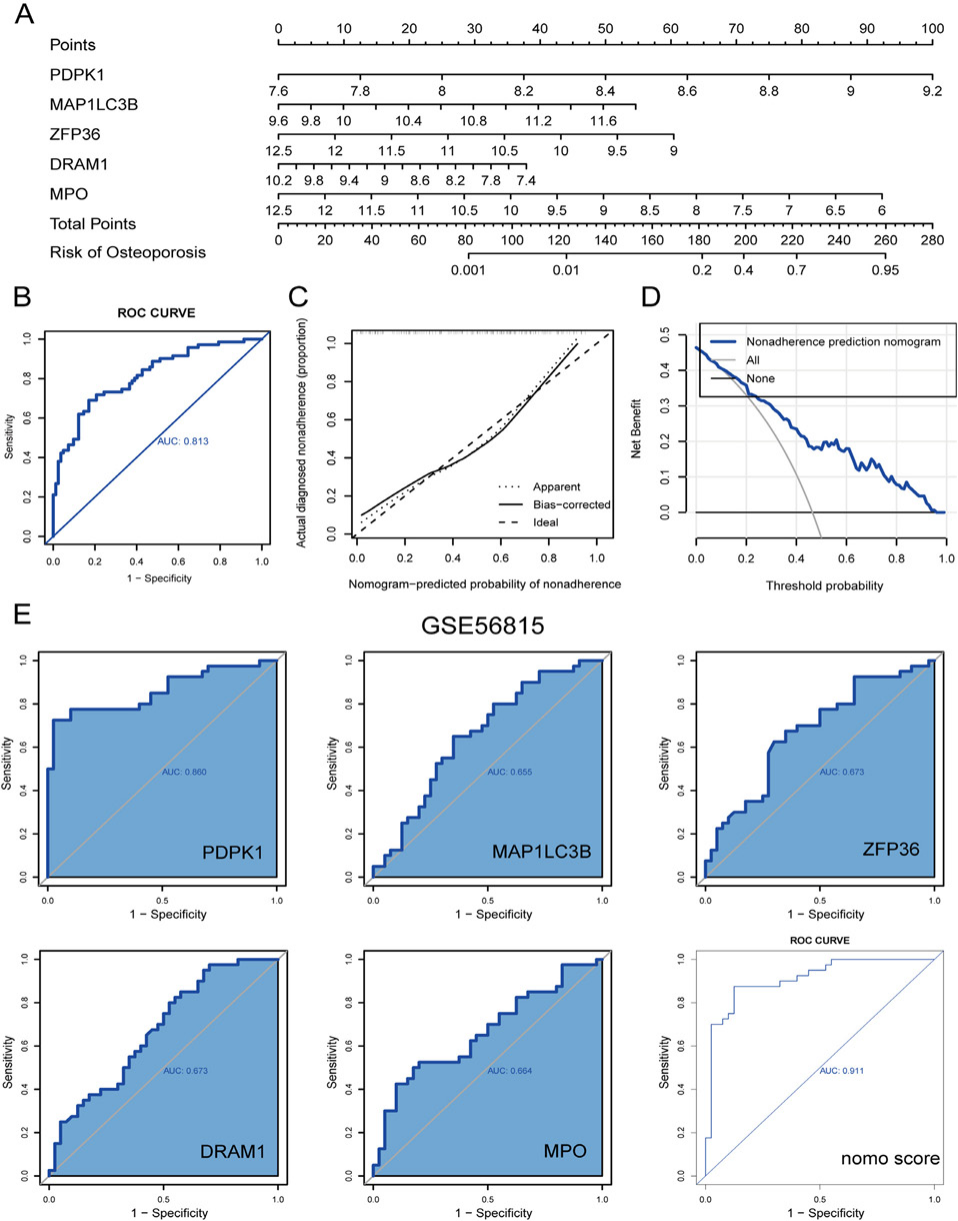
Performance of PCD-related hub genes. (**A**) Developing a nomogram to predict the occurrence of OP using PCD-related hub genes. (**B**) ROC curve of the five hub genes combined diagnosis of OP. (**C**) Calibration curve. (**D**) DCA curve. (**E**) Diagnostic evaluation of hub genes and nomogram score. ROC curve to evaluate prediction accuracy in GSE56815.

A nomogram model was developed using these PCD-related hub genes to assess the risk of developing OP and to further assess their predictive efficacy (Fig. **5A**). A combined ROC analysis of these five hub genes yielded a noteworthy AUC of 0.813 (Fig. **5B**), indicating outstanding diagnostic efficiency. The performance of the nomogram was further confirmed by the calibration curve (Fig. **5C**) and DCA (Fig. **5D**).

In GSE56815, the diagnostic values of the five hub genes (PDPK1, MAP1LC3B, ZFP36, DRAM1, and MPO) and nomogram model scores were assessed using ROC curves. The AUCs for the hub genes were as follows: PDPK1: 0.860, MAP1LC3B: 0.655, ZFP36: 0.673, DRAM1: 0.673, and MPO: 0.664. The nomogram model score recorded an AUC of 0.911 (Fig. **5E**). These findings indicate significant diagnostic relevance of the hub genes for OP. Furthermore, the combined AUC of the nomogram model, based on these genes, exceeded those of any individual gene, indicating that considering a collective approach enhanced the diagnostic accuracy, which is beneficial for predicting clinical outcomes in OP.

### 3.5 Identification of PCD-Related Sub-Clusters in OP and Immune Infiltration Characteristics

**Fig. (6):**
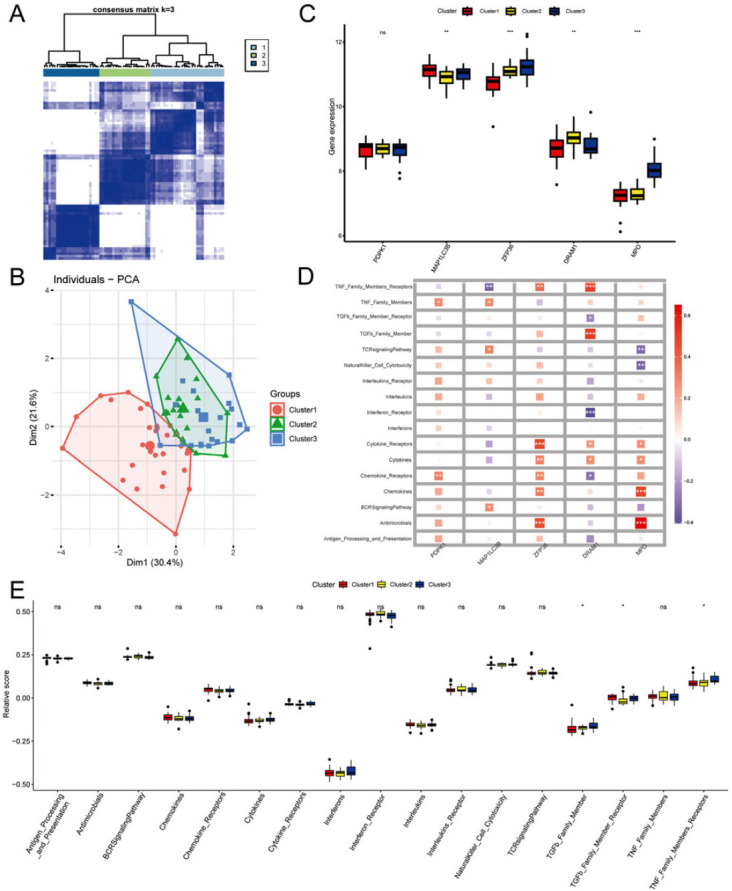
Identification of PCD-related sub-clusters in OP and immune infiltration characteristics. (**A**) Consensus clustering matrix when k = 3. (**B**) PCA was used to verify the three distinct sub-clusters divided by consensus clustering. (**C**) The expression of five hub genes among three sub-clusters. (**D**) Correlation analysis between five hub genes and immune pathways. (**E**) The relative abundances of immune pathways among three sub-clusters.

The R package ConsensusClusterPlus was utilized to establish three sub-clusters (Cluster1, Cluster2, and Cluster3). The analysis revealed that there were 29 samples in Cluster 1, 20 samples in Cluster 2, and 22 samples in Cluster 3. Moreover, it was observed that Cluster 1 can be effectively separated from Cluster 2 and Cluster 3 (Figs. **6A** and **B**). It was observed that except for PDPK1, the remaining 4 hub genes showed significant differences among the three sub- clusters (Fig. **6C** and **6D**). Furthermore, an analysis of the 17 immune-related pathways showed that 3 of the 17 pathways exhibited significant differences among the sub-clusters, including TGF-β family member, TGF-β family member receptor, and TNF family member receptors (Fig. **6E**). These results indicate that inflammatory pathways may have a significant impact on OP development, and these hub genes could potentially regulate immune functions in novel ways.

### 3.6 Functional Enrichment Analysis

GO analysis identified 189 biological processes (BP), 27 cellular components (CC), and 18 molecular functions (MF). As represented in Fig. (**7A**), BP was particularly enriched in response to pyroptosis, lipopolysaccharide (LPS), regulation of autophagy, response to molecule of bacterial origin, and intrinsic apoptotic signaling pathway; CC was significantly enriched in secretory granule lumen, cytoplasmic vesicle lumen, vesicle lumen, azurophil granule lumen, and primary lysosome; MF was mainly enriched in caspase binding, CARD domain binding, C-acyltransferase activity, cytokine binding, and protease binding. The KEGG analysis showed that the candidate DEGs were enriched in NOD-like receptor signaling pathway, Lysosome, NET formation, Kaposi sarcoma-associated herpesvirus infection, Shigellosis, C- type lectin receptor signaling pathway, Neurotrophin signaling pathway, FoxO signaling pathway, Apoptosis, and Lipid and atherosclerosis, as shown in Fig. (**7B**).

**Fig. (7):**
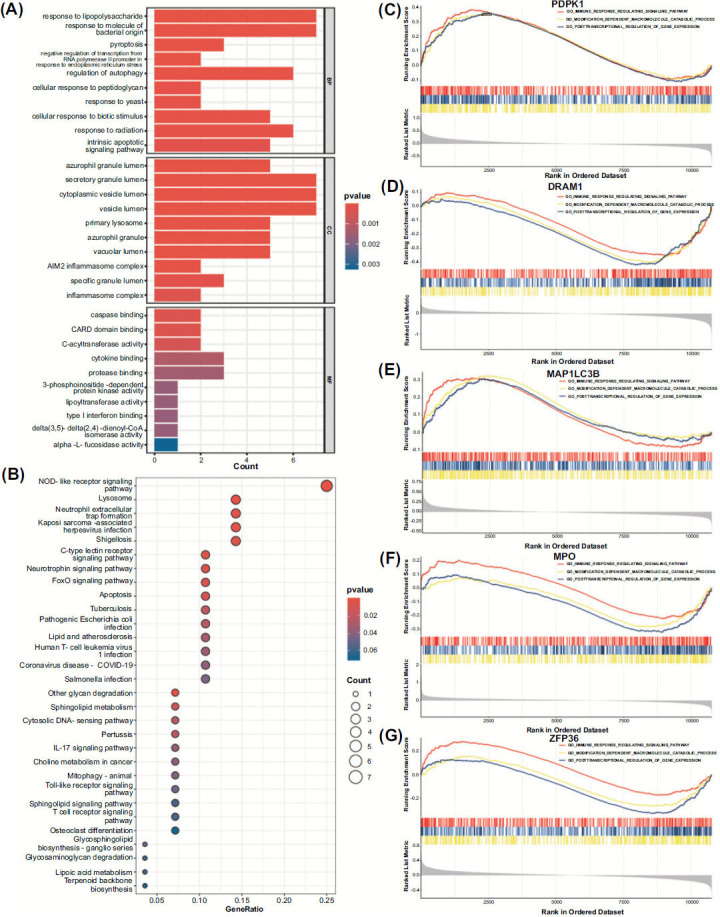
Functional enrichment analysis. (**A**) GO analysis of PCD-related candidate genes for OP. (**B**) Top 30 KEGG pathway analysis of PCD-related candidate genes for OP. (**C**-**G**) GSEA analysis of PCD-related hub genes for OP.

Moreover, to delve into the intricate mechanisms underlying molecular heterogeneity, the GSEA compared the low- expression and high-expression groups, shedding light on key biological processes and pathways. GSEA demonstrated that the hub genes upregulated (PDPK1, MAP1LC3B) and downregulated hub genes (DRAM1, MPO, ZFP36) were primarily enriched in the immune response regulating signaling pathway, modification-dependent macromolecule catabolic process, and post-transcriptional regulation of gene expression (Figs. **7C**-**G**).

## 4 DISCUSSION

OP is a widespread osteodegenerative disorder associated with genetic and environmental risk factors [1], which is believed to stem from an imbalance in bone resorption and synthesis of osteoclasts and osteoblasts [3, 23]. Existing studies have established that PCDs play an indispensable role in both the maintenance and destruction processes of bone homeostasis [44]. Therefore, elucidating the crucial pathways and genetic signatures related to OP and PCD can help with risk assessment and can be instrumental in assessing risk, comprehending disease progression, and tailoring personalized treatment approaches for individuals.

Osteoclasts are the only cells that can absorb bones, and their progenitors are circulating monocytes [45, 46]. Therefore, we focused our analysis on circulating monocyte samples obtained from GEO. In our study, we identified 161 DEGs related to OP, with 114 genes upregulated and 47 genes downregulated. We also identified 30 PCD-related candidate genes associated with OP, as well as five hub genes (PDPK1, MAP1LC3B, ZFP36, DRAM1 and MPO). The candidate genes were further annotated for function enrichment analysis. Additionally, we developed a nomogram using these hub genes to predict OP occurrence.

Among the five hub genes obtained by machine learning, MAP1LC3B, and DRAM1 are closely associated with autophagy, ZFP36 is linked to ferroptosis, PDPK1 is involved in apoptosis and lysosome-dependent cell death, and MPO is directly related to netotic cell death [9]. Notably, there is a crosstalk mechanism between different PCD pathways, such as ferroptosis, which may be a form of autophagy death [47]. Autophagy, crucial for cellular homeostasis [48, 49], plays a pivotal role in regulating bone resorption by controlling osteoclast activation and differentiation [50]. Age-related decline in autophagic activity may exacerbate OP pathogenesis by compromising bone remodeling processes [48, 51]. Additionally, apoptosis, characterized by controlled cellular dismantling and netotic cell death, involving the release of NETs, could impact bone turnover and regulate bone loss [52, 53]. Understanding the intricate interactions between these PCD pathways is crucial for identifying potential therapeutic targets to combat OP-induced bone loss effectively. Further research into these mechanisms may unveil novel strategies for preventing and treating osteoporotic fractures.

MAP1LC3B (microtubule-associated protein 1 light chain 3 beta, LC3-β) is particularly important in autophagy and is essential for cellular homeostasis [54, 55]. DeSelm *et al.* found that knockout of Atg5 in ovariectomized (OVX) mice can prevent experimental postmenopausal osteoporosis by inhibiting the localization of LC3 at the ruffled border [56], suggesting minimizing MAP1LC3B levels as a potential strategy for OP treatment. Similarly, DRAM1 (damage-regulated autophagy modulator 1), another autophagy-related gene [57, 58], affects osteoporosis by promoting autophagy and influencing osteoblast function [59]. Tang *et al.* demonstrated that DRAM overexpression enhances autophagy in osteoblasts, leading to lipid accumulation in OVX mice [60]. Despite reduced autophagy levels in OVX rats, DRAM overexpression significantly promotes autophagy in osteoblasts, inhibiting their proliferation and inducing apoptosis [59].

ZFP36 (ring finger protein 36), encoding Tristetraprolin (TTP), regulates ferroptosis and inflammation by inhibiting autophagic ferritin degradation [45, 61]. Deficiency of TTP in mice elevates systemic inflammation, induces monocyte differentiation into osteoclasts, and causes bone loss [45]. PDPK1 (3-phosphoinositide-dependent protein kinase-1), a participant in PI3K signaling, modulates osteoclast and osteoblast function [62-64]. Previous studies demonstrated that PDPK1 deficiency in osteoclasts protects OVX mice from developing osteoporosis [63, 65]. Additionally, Bai *et al.* showed that PDPK1 knockout in osteoblasts reduces bone mass in mice by inhibiting osteoblast response to IGF-1 signaling [64]. However, further research is required to fully grasp the overall impact of PDPK1 on bone metabolism.

Lastly, MPO (Myeloperoxidase), an inflammation-related enzyme [66], may exert protective effects on bone turnover by modulating osteoblast activity and limiting osteoclast production [67, 68]. Our study's findings on the expression of these hub markers are consistent with previous research, indicating their potential as promising candidates for further investigation and therapeutic targeting in osteoporosis.

In our study, crosstalk genes of PCD and OP primarily participated in biological processes regulated by TGF-β and TNF family member receptors. TGF-β1 can stimulate the proliferation of mesenchymal stem cells, promoting their differentiation into osteoblasts while also affecting osteoclast maturation at lower concentrations [69]. Similarly, TNF super-families, including RANKL, RANK, and OPG, effectively regulate osteoclast formation both *in vitro* and *in vivo* experiments [70, 71]. OPG prevents RANK from binding to RANKL by binding to RANKL itself, thereby inhibiting the formation of osteoclasts and subsequently reducing bone loss [72]. The GO analysis revealed enrichment in pathways mostly associated with responses to LPS, response to molecules of bacterial origin, autophagy regulation, and intrinsic apoptosis signaling. Indeed, the response to LPS, known to induce apoptosis in osteoblasts and osteocytes, has been associated with aggravated bone loss, potentially contributing to osteoporosis [73]. Moreover, Pyroptosis triggered by bacterial infection promotes osteomyelitis by inhibiting osteoblasts and activating osteoclasts, potentially contributing to the onset and progression of osteoporosis [74]. Furthermore, the KEGG analysis revealed pathways like NOD-like receptor signaling, NET formation, and apoptosis, implicated in chronic inflammation, immune dysregulation, and disrupted bone homeostasis, driving osteoporosis [52, 53, 75]. NOD-like receptor-mediated pyroptosis may worsen osteoporosis by hyperactivating osteoclasts [75]. These pathways significantly influence bone remodeling, impacting osteoclastogenesis, osteoblast function, and bone turnover, showcasing the complex interplay between programmed cell death mechanisms and bone metabolism in osteoporosis development.

The correlation analysis revealed a robust and statistically significant positive correlation (all > 0.6, except MPO:0.594). Hence, it is hypothesized that they are critical to the pathology of OP. Additionally, a nomogram model was developed to predict the risk of OP. At present, the diagnosis of OP mainly relies on DXA examination, but the prevalence rate is not enough in people without fractures. Accurate diagnosis and early precautions for OP are vital in minimizing suffering and improving the condition's prognosis. In the GSE56815 dataset for OP, the AUCs for the five hub genes exceeded 0.65, indicating that they possess strong diagnostic values. Additionally, the nomogram model achieved an AUC of 0.911 (GSE56815), demonstrating high accuracy in predicting the disease. Overall, these findings suggest that PDPK1, MAP1LC3B, ZFP36, DRAM1, and MPO, and the nomogram model based on these genes hold promise as diagnostic biomarkers and could serve as potential therapeutic targets for OP.

However, this study has limitations. Firstly, the sample tissues originated from circulating monocytes instead of bone tissues, which prevents us from confirming the suitability of the selected diagnostic markers for bone tissue measurement. Additionally, our findings were based solely on bioinformatics analysis. Further *in vivo*, *in vitro*, and clinical studies are necessary to validate these results. Moreover, the small sample sizes in some sub-clusters, due to the limitations of the public databases used, may affect the robustness and generalizability of our results. Future research should incorporate larger sample sizes to validate these findings more effectively. Lastly, this research was limited to gene expression data alone. Future research endeavors should consider epigenetic, metabolomic, and proteomic variations to deepen our understanding of OP pathogenesis.

## CONCLUSION

Through bioinformatics and machine learning, this research investigated the molecular characteristics of PCD in OP and identified five potential biomarkers: PDPK1, MAP1LC3B, ZFP36, DRAM1, and MPO. Our findings suggested that PCD mechanisms such as autophagy, apoptosis, and immune-related pathways play critical roles in OP development. Additionally, a predictive nomogram incorporating these biomarkers was developed for the clinical diagnosis of OP. These biomarkers may offer new directions for both diagnostic and therapeutic strategies, although further experimental validation is needed to confirm their clinical utility.

## Figures and Tables

**Fig. (1):**
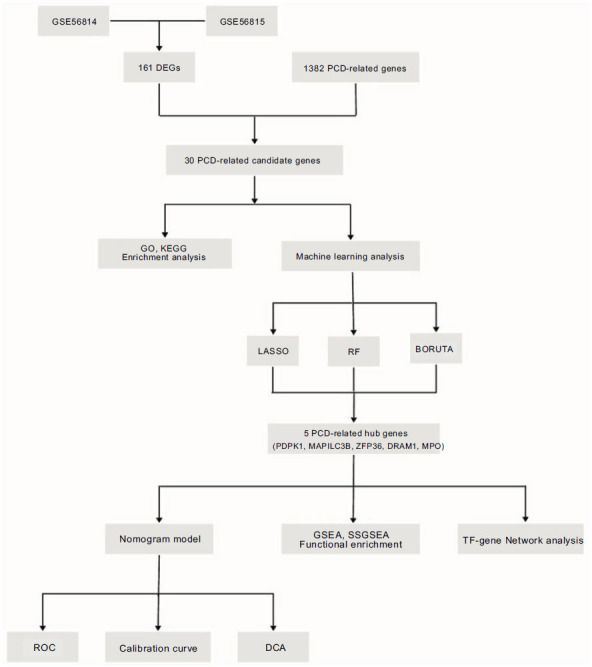
The study’s flow diagram.

## Data Availability

All data generated or analysed during this study are included in this published article.

## LIST OF ABBREVIATIONS

OP: Osteoporosis
PCD: Programmed Cell Death
Degs: Differentially Expressed Genes
LASSO: Least Absolute Shrinkage And Selection Operator
RF: Random Forest
BMD: Bone Mineral Density
DXA: Dual-Energy X-Ray Absorptiometry
Sds: Standard Deviations
ACD: Accidental Cell Death
NET: Neutrophil Extracellular Trap
Bmscs: Bone Mesenchymal Stem Cells
GEO: Gene Expression Omnibus
FC: Fold Change
TF: Transcription Factors
ROC: Receiver Operating Characteristics
AUC: Area Under The Curve
DCA: Decision Curve Analysis
Ssgsea: Single Sample Gene Set Enrichment Analysis
GO: Gene Ontology
KEGG: Kyoto Encyclopedia Of Genes And Genomes
GSEA: Gene Set Enrichment Analysis
Msigdb: Molecular Signature Database
BP: Biological Processes
CC: Cellular Components
MF: Molecular Functions
LPS: Lipopolysaccharide
MAP1LC3B/ LC3-Β: Microtubule-Associated Protein 1 Light Chain 3 Beta
DRAM1: Damage-Regulated Autophagy Modulator 1
ZFP36: Ring Finger Protein 36
TTP: Tristetraprolin
PDPK1: 3-Phosphoinositide-Dependent Protein Kinase-1
MPO: Myeloperoxidase
OVX: Ovariectomized
